# Aberrant Right Subclavian Arteries in Dogs Have a Prevalence of 1.2% and Are More Likely to be an Incidental Finding on Computed Tomographic Studies of the Thorax

**DOI:** 10.1111/vru.70011

**Published:** 2025-02-09

**Authors:** Coleen Jones, Julius Klever, Alessia Cordella, Virginie Fouriez‐Lablée, Thom C. Watton, Francisco Llabres‐Diaz

**Affiliations:** ^1^ Department of Clinical Science and Services Royal Veterinary College Hatfield UK; ^2^ Department of Clinical Sciences and Advanced Medicine Section of Radiology University of Pennsylvania School of Veterinary Medicine Philadelphia Pennsylvania USA; ^3^ VET.CT, Hauser Forum, The Broers Building Cambridge Cambridgeshire UK; ^4^ 7 Follaton Totnes Devon UK

**Keywords:** anatomical variant, arteria lusoria, retroesophageal right subclavian artery, vascular anomaly

## Abstract

Aberrant right subclavian arteries (ARSAs) are a form of vascular ring anomaly (VRA) in dogs and the most common VRA in people. To date, there has been no large‐scale study on ARSA in dogs and their potential clinical significance. For part one, a single‐center retrospective observational study was performed to determine the prevalence of ARSAs in a population of 1000 dogs undergoing contrast‐enhanced CT for various reasons. For part two, further canine ARSA cases were collected to characterize their imaging features further and determine whether any imaging findings were more frequent in dogs with clinical signs attributed to a VRA. The prevalence of ARSA was 1.2% (12/1000). For part two, out of a total of 37 dogs with ARSA, this finding was thought to be incidental in 28 cases (75.6%), clinically relevant in 1 case (2.7%), and potentially relevant in 8 cases (21.6%). Cranial esophageal dilation with gas and fluid and esophageal compression at the site of the ARSA crossing the esophagus was found in the case where the ARSA was considered relevant. Esophageal dilation with esophageal compression by the ARSA was also more frequent in the potentially relevant group. Our study shows that an ARSA is more likely to be an incidental finding; however, due to the low number of cases where the ARSA was considered relevant, no specific imaging findings were found that could help determine their clinical relevance.

## 1 Introduction

An aberrant right subclavian artery (ARSA) is a type of vascular ring anomaly (VRA) in dogs and can occur with or without a concurrent persistent right ligamentum arteriosum [1, 2, 3, 4]. Usually, an ARSA is the last vessel to exit the aortic arch, distal and dorsal to the common carotid arteries and normal left subclavian artery. The ARSA then passes dorsally and medially to the right of the aortic arch and in a cephalad direction. This means the ARSA passes dorsal to the trachea and esophagus, forming a partial ring around these structures [3, 4]. In some papers, an ARSA is referred to as an aberrant retroesophageal right subclavian artery as it passes behind the esophagus and trachea [2]. Previously, nine different types of vascular ring anomaly have been described [1, 2, 3, 4, 5]; however, recently, a case series has described a left circumflex aortic arch in three dogs, one of which also had an ARSA [6].

Clinical signs associated with a VRA commonly occur after weaning and include regurgitation, dysphagia, failure to gain weight or weight loss, and subsequently poor body condition [3, 4, 5]. These effects are caused by varying degrees of esophageal constriction by the anomalous vessel or ligament, inhibiting the transport of food and fluid boluses to the stomach. Frequent regurgitation can lead to aspiration pneumonia in these patients [5]. Tracheal deviation, and sometimes tracheal compression, have been described in patients with VRAs [7, 8, 9]. Clinical signs associated with the effects on the trachea have been reported, although these signs are not always apparent and do not seem to correlate with the degree of tracheal compression [7]. Plain radiography, esophageal contrast studies, selective angiography, and CT can all be used to diagnose VRAs [8, 10]. Dilation of the cranial thoracic esophagus with focal narrowing cranial to the heart base is a common finding; however, some VRAs cause minimal or no changes on plain radiography, positive contrast esophagography, or videofluoroscopy [5, 10, 11]. The use of multidetector CT (MDCT) has improved the diagnosis of VRAs due to the ability to clearly visualize the major blood vessels and create multiplanar and 3D reconstructions [12, 13].

In two recent papers, the prevalence of ARSA in cases undergoing MDCT was 0.8% and 1.6% [1, 2]. In the paper by Schorn et al. (2021), three dogs had an aberrant right subclavian artery, and none of these dogs had clinical signs that could be attributed to a VRA [1]. An anatomical case series [14] and a case report [15] describe the post‐mortem finding of an ARSA, of which no dog had a history of clinical signs compatible with a VRA antemortem. There are also case reports of dogs with an ARSA that have clinical signs compatible with a VRA, such as regurgitation, weight loss, and poor body condition. These included young dogs, as well as adult dogs with new onset clinical signs. Some cases also had features compatible with aspiration pneumopathy [11, 16–21]. Both surgical (ligation of the ARSA) and medical (dietary modification) management have proved to be effective in these cases [18, 19, 20].

ARSAs are the most common VRA in humans, with a reported prevalence of 0.4–1.8% [22, 23, 24]. People with ARSA are usually asymptomatic, but approximately 5% of them, often in later life, may present with clinical signs, including dysphagia, dyspnea, cough, chest pain, and claudication of the right upper limb [22, 23, 24]. It is also an important consideration prior to procedures involving or adjacent to the esophagus where inadvertent damage to the vessel could lead to major bleeding [22].

At our institution, it was noted that ARSAs were variably detected in cross‐sectional imaging studies and that most of the cases had no clinical signs that could be related to a VRA. To the authors’ knowledge, there have been no studies on a larger population of dogs with ARSAs, and previous studies suggest that further research with larger numbers is warranted [1, 2, 3, 4]. As CT becomes more widely available, it is crucial to help users discriminate between potentially significant findings and those that are most likely to be incidental, especially if more than one potential cause of a clinical sign is found.

With this two‐part study, we aimed to, first, determine the prevalence of (1) ARSAs and other thoracic vascular anomalies and anatomical variations, (2) esophageal dilation, and (3) clinical signs of regurgitation within our hospital population undergoing thoracic CT. Second, we aimed to describe key imaging findings with relation to the ARSA, esophagus, and trachea in patients with an ARSA to attempt to find differences between cases in which the ARSA was most likely an incidental finding versus those in which the ARSA was deemed clinically significant.

We had the following hypotheses:

‐ The prevalence of ARSA would be higher than previously published.
‐ The number of dogs with ARSA and clinical signs that could be explained by a VRA would be low.
‐ The pathway of the ARSA and the appearance of the esophagus might differ between incidental and nonincidental cases.

## 2 Materials and Methods

### 2.1 Study Design and Case Selection

A retrospective observational two‐part study was performed following the STROBE guidelines. Part one was a cross‐sectional prevalence study, and part two was a descriptive case series design. Both parts were performed using clinical and imaging data from the Royal Veterinary College (RVC). Due to the retrospective nature of the study, no ethical approval was required. For part one, the CT imaging log was searched for dogs undergoing a minimum of comparable pre‐ and postcontrast thoracic CT. Cases were continuously collected from December 16, 2022, backward until 1000 dogs met the inclusion criteria (July 1, 2021). Duplicate cases due to multiple CTs of the same animal were excluded.

The inclusion criteria were as follows: A diagnostic quality CT of the thorax, including a postcontrast phase, and a complete clinical history available for review. Patients were excluded if the images were not of sufficient quality to assess the thoracic vasculature. Patients were classified as having an ARSA if there was no normal right subclavian artery arising from the brachiocephalic trunk and an anomalous vessel arose from a normal left aortic arch with a path toward the first rib on the right, that is, a type 6 or 7 VRA [1]. Patients with other types of vascular ring anomaly were included in the prevalence study as part of the no ARSA (nARSA) group and described within the results section. These cases were excluded from further statistical analysis. Other vascular anomalies or variations in the thoracic vasculature were classified as previously described [1, 2].

For part 2, the RVC's Vetcompass database was searched for other cases with aberrant right subclavian arteries in dogs treated at RVC between July 2018 and December 2022 (cases seen prior to July 2018 were not included as different CT equipment was used). The term “subclavian” was used when searching the database. Patients were then included if they had a confirmed ARSA based on computed tomographic diagnosis, diagnostic quality CT images available for review, and a complete clinical history. Cases that were found during the prevalence study were also included in this part of the study.

### 2.2 Data Recording

For part 1, the following data were recorded by a first‐year European College of Veterinary Diagnostic Imaging resident (C. J.): age, sex, breed, weight, method of restraint (sedation, general anesthesia, or consciousness), overall disease categorization, presence or absence of a history of regurgitation. Overall, disease categorization was based on the history, problem list, and final diagnosis. The categories included neoplastic, neurological, surgical, surgically treated brachycephalic obstructive airway syndrome (BOAS), gastrointestinal, hematological, respiratory (upper, lower, or combined upper and lower) acquired and congenital cardiac disease, and miscellaneous (which included hepatic disease, ocular disease, immune‐mediated disease, orthopedic disease, multifactorial disease or any other disease category not listed), similar to a previous study [2].

For part two, the same data was recorded, as well as the clinical problem list and final diagnosis, including any findings consistent with aspiration pneumopathy. If the ARSA was or was not recorded in the imaging report, and if any specific management was put in place due to the finding of an ARSA in the discharge letter, this was recorded. If other imaging studies were performed, the results of these studies were included. If follow‐up was available, this was assessed, and any change in the frequency of regurgitation was recorded.

### 2.3 CT Image Analysis

For part 1, the CT scans were retrieved and interpreted by one radiologist (F. L. D., J. K., A. C., V. F. L., and T. W.) and the ECVDI resident (C.J.) using DICOM viewing software (eUnity v.7.3.2‐334, Mach7, South Burlington, VT). All the reviewers were provided with instructions regarding the necessary information to be collected for the study. Multiplanar reformatting was used as needed for interpretation. Images were viewed in a smooth reconstruction algorithm using a soft tissue window (window width of 400HU and window level of 40).

**FIGURE 1:**
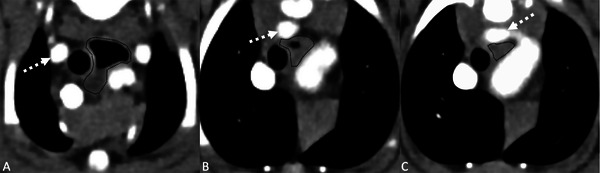
Postcontrast transverse CT images in a soft tissue window (WW 400, WL 40) showing a representative example of esophageal dilation (A) and compression (B, C) in a patient with an aberrant right subclavian artery (ARSA, depicted with the dashed arrow). Left is to the right of the image. (A) Cranial to the heart base the esophagus (encircled by the dashed black line) is dilated with gas dorsally, and fluid attenuating content ventrally. (B, C) As the ARSA passes dorsally, the esophageal diameter is markedly reduced compared with image (A). This patient had clinical signs attributable to the ARSA.

Images were assessed for diagnostic quality, the presence or absence of an ARSA originating from a normal left aortic arch, the classification of the common carotid arteries [2], and the presence or absence of other thoracic vascular abnormalities. For cases in sternal recumbency only, the presence of oesophageal dilation was assessed. Esophageal dilation was categorized as present if the esophagus was dilated to a greater diameter than the patient's trachea at any point along its length within the thorax (Figure 1).

For part 2, the following additional data were collected and agreed upon based on consensus between three authors (C. J., J. K., and F. L. D.): the order in which the vessels arose from the aortic arch (from cranial to caudal), the description of the path of the aberrant right subclavian artery, any focal dilatation of the aberrant vessel, any sharp/ angular change in the direction of the vessel, the rib spaces at which the ARSA traversed the esophagus and trachea, the subjective presence or absence of any tracheal compression or deviation, and the presence of any oesophageal dilation or compression. Oesophageal dilation was assessed similarly to part one but was split into two regions, cranial and caudal, to the ARSA. The location and path of the descending aorta were assessed and categorized as normal if it was to the left of the midline and abnormal if it was on the midline or to the right of the midline.

ARSAs were put into categories based on the presence or absence of a history of regurgitation or a diagnosis of aspiration pneumopathy with no other cause identified. The ARSA was categorized as most likely incidental if there was no history of regurgitation and no finding of aspiration pneumopathy. The ARSA was categorized as potentially relevant if there was a history of regurgitation and/ or findings of aspiration pneumopathy. If a patient with an ARSA had any of the above features and specific intervention was performed due to the finding of an ARSA, after which the patient's clinical signs improved, then this ARSA was recategorized as clinically relevant. Regurgitation and aspiration pneumopathy were used as they were considered the most specific clinical features that could influence the radiologist's conclusion about the finding of an ARSA.

### 2.4 Statistical Analysis

All data were entered into a spreadsheet (Excel for Windows, Microsoft, version 2403 Build 16.0.17425.20176), and descriptive statistical analyses were performed by two authors (C. J. and F. L. D,) with formal training in applied statistics using SPSS (SPSS IBM, New York, NY, version 29.0.0.0 (241)). Normality was assessed with a visual assessment of histograms and the Kolmogorov‐ Smirnov test. Where one group was parametric, and another was nonparametric, both were presented as median (range). The Fisher's exact test was used for analysis between categorical variables. The Mann–Whitney *U* test was used to compare continuous, nonparametric data between groups. A *p*‐value of .05 was considered statistically significant for these analyses.

## 3 Results

### 3.1 Imaging Techniques

The choice of sedation or anesthesia and the drugs used was based on the clinical judgment of the primary clinicians or anesthetists. For part one, the CT images were acquired in sternal recumbency in 993 dogs (99.3%), lateral recumbency in 6 dogs (0.6%), and dorsal recumbency in one dog (0.1%). All CT images for the cases in part two were acquired in sternal recumbency. Sternal recumbency was used as standard, with other recumbencies used based on the clinical history and presenting clinical signs. For both parts of the study, images were acquired with a 320‐slice CT scanner (Aquilion ONE‐Genesis, Canon Medical Systems, Japan) before and after contrast (2 mL/kg; Omnipaque, 300 mg iodine/mL, GE Healthcare, UK) administration via an intravenous catheter using a pressure injector (Medrad Stellant CT dual Injection System, Bayer Medical Care B.V., the Netherlands). The following settings were used: 120 kVp, slice thickness 2–3 mm, 0.8–1.4 pitch, matrix size 512 × 512, a soft tissue/smooth reconstruction algorithm, and image field‐of‐view tailored individually. The mAs was individually modulated using ^SURE^Exposure software (Canon Medical Systems, Otawara, Japan). The contrast flow rates were adjusted according to the body weight. All Images were obtained from cranial to caudal; a 45–70 s delayed (from contrast injection initiation) postcontrast phase was acquired as standard, with other contrast phases acquired on a case‐by‐case basis.

### 3.2 Part One

#### 3.2.1 Prevalence Study

For part one, 1001 cases were assessed, and one was excluded due to incomplete clinical records. Therefore, 1000 dogs were included over the 17.5 months. The CT studies were performed under general anesthesia in 525 dogs (52.5%), under sedation in 474 (47.4%), and conscious in 1 (0.1%). Twelve dogs had an ARSA (1.2%, CI 95% (0.6–2.1%)). Data regarding the age, weight, sex, overall disease categorization, and other vascular anomalies and anatomical variants are presented in Table 1 for the dogs with (part one and part two) and without (nARSA) an ARSA. Data regarding the breeds represented in each group are shown in Table 2 (part one only).

**TABLE 1:** Descriptive data including age, weight, sex, overall disease categorization, and other vascular anomalies and anatomical variants for parts one and two of the study.

	Part one		Part two
	ARSA (12)	nARSA (988)	ARSA (37)
Age: median (range)	5 years (10 months–11 years 6 months)	8 years, 5 months (3 months–17 years)	6 years, 8 months (2 months–12 years, 4 months)
Weight (kg)	Median 18.6 (range 8.7–43)	Median 18.6 (range 1.5–80)	Mean 21.7 (SD ±11.2)
Sex			
Male	11 (91.7%)*	575 (58.2%)	26 (70.3%)
Female	1 (8.3%)	413 (41.8%)	11 (29.7%)
Overall disease categorization			
Neoplastic	5 (41.7%)	410 (41.5%)	11 (28.7%)
Miscellaneous	5 (41.7%)	212 (21.5%)	15 (40.5%)^a^
Surgically treated BOAS	2 (16.7%)	78 (7.9%)	3 (8.1%)
Surgical	0	71 (7.2%)	0
Lower respiratory	0	50 (5.1%)	4 (10.8%)
Hematological	0	48 (4.9%)	0
Neurological	0	46 (4.7%)	2 (5%)
Upper and lower respiratory	0	19 (1.9%)	1 (2.7%)
Gastrointestinal	0	19 (1.9%)	1 (2.7%)
Upper respiratory	0	18(1.8%	0
Acquired cardiac	0	16 (1.6%)	0
Congenital cardiac	0	1 (0.1%)	0^a^
Other vascular anomalies and anatomical variants			
Bicarotid trunk	12 (100%)	55 (5.6%)	37 (100%)
PRAA and aberrant left subclavian artery	0	2 (0.2%)^b^	0
Persistent left cranial vena cava	0	13 (1.3%)	0
Extrahepatic portoazygous shunt with dilation of the thoracic azygous vein	0	3 (0.3%)	0
Azygous continuation of the caudal vena cava and malformation of the caudal vena cava	0	1 (0.1%)	0
Undetermined arteriovenous malformation between the azygous vein and the pulmonary artery or aorta	0	1 (0.1%)	0
Intrathoracic collateral venous circulation due to cranial vena cava pathology	0	1 (0.1%)	0
Situs inversus	0	1 (0.1%)	0

Abbreviations: ARSA, aberrant right subclavian artery group; nARSA, no aberrant right subclavian artery; PRAA, persistent right aortic arch; BOAS, brachycephalic obstructive airway syndrome.

* *p *< .05.

^a^ One case presented for BOAS also had multiple congenital cardiac abnormalities and was categorized as miscellaneous.

^b^ One case had clinical signs of a VRA and had a surgically confirmed persistent ligamentum arteriosum. In the other case, it was considered an incidental finding and no surgery was performed.

**TABLE 2:** Relative prevalence of aberrant right subclavian artery (ARSA) per breed, expressed as a percentage, included in part one of the study.

Breed	ARSA (12)	nARSA (988)	Total	Prevalence of ARSA per breed
Crossbreed	1 (8.3%)	234 (23.7%)	235	0.4%
Labrador Retriever	1 (8.3%)	84 (8.5%	85	1.2%
French Bulldog	3 (25%)	80 (8.1%)	83	3.6%
Cocker Spaniel	1 (8.3%)	52 (5.2%)	53	1.9%
German Shepherd	1 (8.3%)	27 (2.7%)	28	3.57%
English Bulldog	1 (8.3%)	23 (2.3%)	24	4.2%
Boxer	1 (8.3%)	10 (1.0%)	11	9.1%
English Setter	1 (8.3%)	2 (2.0%)	3	33.3%
Scottish Terrier	2 (16.7%)	0	2	100%
Staffordshire Bull Terrier	0	29 (2.9%)	29	0%
Golden Retriever	0	28 (2.8%)	28	0%
Other breeds^a^	0	447 (45.2%)	447	0%

Abbreviations: ARSA, aberrant right subclavian artery group; nARSA, no aberrant right subclavian artery.

^a^ Other breeds with 27 or fewer within the group and without an ARSA. A total of 91 different breeds are represented in this row.

Male dogs were overrepresented in the ARSA group, and this finding was statistically significant (*p* =  .019). There was no significant difference between the two groups for age, weight, or disease categorization (*p* =  .061, .381, and .866, respectively). Differences between breeds were not statistically assessed due to the relatively small number of breeds with an ARSA and large variation between breed group sizes.

#### 3.2.2 Regurgitation

The total prevalence of regurgitation was 9.9% (99/1000). In the ARSA group, two dogs (2/12, 16.7%) had a history of regurgitation; both presented for BOAS. Excluding the two patients with PRAA and aberrant left subclavian arteries, 96/986 (9.7%) dogs in the nARSA group had a history of regurgitation, and 890 (90.3%) did not. 44 of 96 (45.8%) dogs in the nARSA group with regurgitation were presented for BOAS.

#### 3.2.3 Esophageal dilation

When assessing esophageal dilation, the two cases with PRAA and those not in sternal recumbency (*n* = 7) were excluded from statistical analysis. The total prevalence of esophageal dilation was 14.7% (146/991), with one case (9.1%) in the ARSA group and 145 cases (14.8%) in the nARSA group.

### 3.3 Part Two—Findings in Dogs With ARSAs

For part two, 41 cases with an ARSA were found; four cases were excluded as only magnetic resonance images were available. Therefore, a total of 37 cases were included. For the dogs included in part two, 25 of 37 (67.6%) were performed under general anesthetic, and 12 of 37 (32.4%) were performed under sedation. Data regarding the age, sex, weight, overall disease categorization, and any other thoracic and vascular anomalies are presented in Table 1. More common breeds included French Bulldogs (*n* = 6, 16.2%), crossbreeds (*n* = 5, 13.5%), Cocker Spaniels (*n* = 4, 10.8%), followed by German Shepherd Dogs and Boxers (*n* = 3 each, 8.1%), Labrador retriever, Irish Setter, Scottish Terrier and Springer Spaniel (*n* = 2 each, 5.4%), and Border Collie, American Bulldog, Chow Chow, Flat‐coated Retriever, English Setter, Boston Terrier, Dalmatian and English Bulldog (*n* = 1 each, 2.7%).

In one case (2.7%), the ARSA was specifically discussed in the discharge letter, and there was a specific change in management post‐CT. In eight cases (21.6%) the ARSA was not mentioned in the imaging report or the discharge letter (these cases were found in part one of the study). In the remaining cases (*n* = 28, 75.6%), the ARSA was reported in the imaging report and not specifically mentioned in the discharge letter.

**FIGURE 2:**
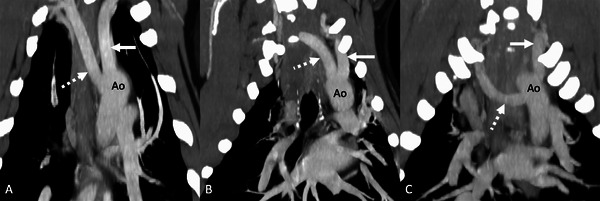
Postcontrast dorsal planar maximum intensity projection CT images showing the different origins of the aberrant right subclavian artery (ARSA, depicted with the dashed white arrow) seen in the current study in a soft tissue window (WW 400, WL 40). Left is to the right of the image. (A) The ARSA originates just caudal and medial to the left subclavian artery (LSA, depicted by the solid white arrow). (B) The ARSA appears to share the same ostia with the LSA. (C) The ARSA originated far caudal and medial to the LSA. The ARSA in all of these cases was considered incidental. Ao, aorta.

**FIGURE 3:**
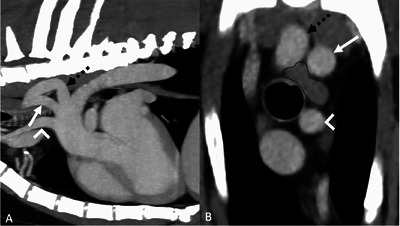
CT images of a dog with an aberrant right subclavian artery (ARSA, depicted by the dashed black arrow) with an acute angular change in direction close to its origin (WW 400, WL 40). The left subclavian artery is shown by the solid white arrow, and the bicarotid trunk is shown by the white arrowhead. (A) Postcontrast sagittal multiplanar reconstruction, maximum intensity projection. (B) Postcontrast transverse image at the level where the ARSA crossed dorsally over the esophagus (encircled by the dotted black line). In this case, the ARSA was considered most likely incidental, and there was no history of regurgitation at the initial presentation or at the follow‐up examination.

**FIGURE 4:**
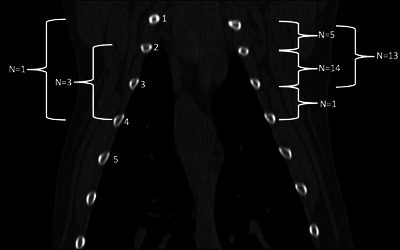
Precontrast dorsal multiplanar reconstruction CT image of a canine thorax, showing the variation in the points at which the aberrant right subclavian artery (ARSA) crossed the esophagus (bone window). The ribs are numbered to the left side of the image, and the brackets represent the length of the esophageal crossing. The number of patients with that type of crossing are indicated next to the bracket (WW 1500, WL 300).

The key imaging features are presented in Table 3, separated into the categories of clinically relevant, potentially clinically relevant, and most likely incidental. In all (100%) cases, the order of vessels originating from the aortic arch, from cranial to caudal, was a bicarotid trunk (a short length of common vessel prior to the bifurcation into the common carotid arteries), left subclavian artery, and then ARSA. All cases also had a normally positioned descending aorta. Three different origins of the ARSA were found, shown in Figure 2. No ARSA had a focal dilatation at its origin. All ARSA passed dorsally over the trachea and esophagus. In 31 cases (83.8%), the ARSA passed initially in a medial, cranial, and dorsal direction. In five cases (13.5%), the ARSA passed dorsomedially before turning cranially. In one case (2.7%), the ARSA passed dorsocaudally and medially initially before turning cranially. From this point, all ARSA passed cranially and slightly ventral to reach the level of the first rib. An acute angular change in the direction of the ARSA was seen in two cases (5.4%) close to the vessel's origin (Figure 3). In both these cases, the ARSA was considered incidental. The ARSA had some variation in the point at which they crossed the esophagus, as shown in Figure 4.

**TABLE 3:** Key Imaging and signalment features in patients with an aberrant right subclavian artery, divided into cases where the ARSA was considered to be clinically relevant, potentially relevant, or most likely incidental.

		Relevant cases		Potentially relevant cases		Most likely incidental cases	
		Number	Percentage (%)	Number	Percentage (%)	Number	Percentage (%)
Imaging feature	Total	1/37	2.7	8/37	21.6	28/37	75.7
Origin of the ARSA	ARSA just caudal to LSA	0/1	0	8/8	100	23/28	82.1
	ARSA and LSA share the same ostia	0/1	0	0/8	0	4/28	14.3
	ARSA originating far caudal to the LSA	1/1	100	0/8	0	1/28	3.6
Esophageal dilation^a^		1/1	100	4/8	50	7/28	25
	Cranial to the heart base only	1/1	100	1/8	12.5	1/28	3.6
	Caudal to the heart base only	0/1	0	1/8	12.5	3/28	10.7
	Cranial and caudal to the heart base	0/1	0	2/8	25	3/28	10.7
Esophageal compression^b^		1/1	100	4/8	50	6/28	21.4
Compatible with aspiration pneumopathy		0/1	0	3/8	37.5	0/28	0
Relevant history or signalment feature							
Regurgitation		1/1	100	6/8	75	0/28	0
Brachycephalic breed		1/1	100	5/8	62.5	5/28	17.9
	Of which presented for treatment of BOAS	0/1	0	4/5^c^	80^c^	0/5	0

Abbreviations: ARSA, aberrant right subclavian artery group; LSA, left subclavian artery; BOAS, brachycephalic obstructive airway syndrome.

^a^ Esophageal dilation with any content, making the diameter greater than the trachea of the same patient.

^b^ Marked reduction in diameter of the esophagus at the level of the ARSA crossing dorsally over the esophagus.

^c^ One of these dogs had a concurrent history of congenital heart disease.

**FIGURE 5:**
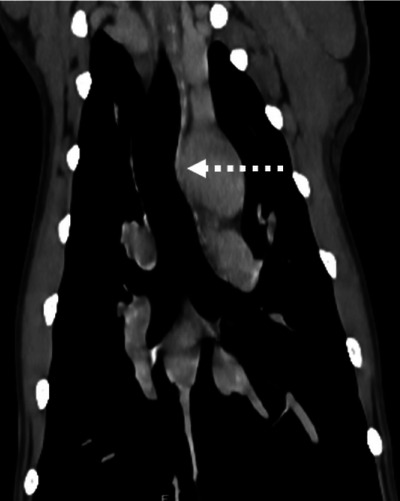
Postcontrast dorsal maximum intensity projection CT image of a dog showing an exaggerated rightward deviation of the trachea at the level of the origin of the aberrant right subclavian artery (white dashed arrow). WW 400, WL 40, slice thickness 3 mm.

When assessing any potential effects on the trachea, four cases could not be assessed due to the presence of an endotracheal tube at the level where the ARSA crossed. The trachea was minimally compressed in one case (1/33, 3%), and no compression was seen in the remaining cases (32/33, 97%). The trachea had a more exaggerated rightward deviation than expected at the level of the ARSA in 5 of 33 cases (15.2%; Figure 5). In one of these dogs, other intrathoracic pathology and conformation findings were likely contributing to this change. In the remaining cases, no tracheal deviation was seen (28/33, 84.8%). No tracheal compression or deviation was seen in the relevant or potentially relevant ARSA cases.

In the relevant case, the patient had a positive contrast videofluoroscopic swallowing study performed as part of the initial investigations prior to the CT scan, revealing a repeatable focal dorsal extramural esophageal narrowing cranial to the heart base. However, food and fluid boluses successfully passed this region, and no other abnormalities were detected. No further imaging studies were available for the other cases.

Follow‐up was available in 17 cases (varying from 9 to 932 days). In the clinically relevant case, the patient underwent surgical ligation of the ARSA. At 235 days post‐CT, the frequency of regurgitation had reduced from daily regurgitation after eating to once monthly regurgitation. Two cases with lower respiratory disease and a history of regurgitation, where the ARSA was considered potentially relevant, died in hospital after cardiopulmonary arrest within two days of the CT scan (from the discharge letter, the reason for death was a severe septic process in one case and septic shock and multiple organ dysfunction in one case). Out of the two other cases categorized as respiratory (one lower respiratory only, one upper and lower respiratory) without a history of regurgitation, one case had no follow‐up available and had no reported regurgitation 932 days post‐CT. Out of the remaining potentially relevant cases, the three categorized as surgical BOAS had no follow‐up available, and the one categorized as miscellaneous continued to regurgitate every 2–3 days at 490 days post‐CT. Follow‐up was available in 14 cases where the ARSA was considered an incidental finding, ranging from 9 to 640 days (median 65.5 days). No regurgitation was noted. Three cases were euthanized during hospitalization for reasons unrelated to an ARSA. In 15 cases, no follow‐up was available. Information regarding the clinically relevant and potentially relevant cases is summarized in Table 4.

**TABLE 4:** Key findings in patients with an aberrant right subclavian artery, where the vascular anomaly was considered relevant or potentially relevant to the patient.

Signalment	Origin of ARSA	Esophageal crossing point (intercostal spaces)	Esophageal dilation	Esophageal compression (Yes/No)	Problem list	Diagnosis	Follow‐ up	ARSA clinically relevant (CR) or potentially relevant (PR) to the patient
2 months, male, Boston Terrier	3.6 mm caudal and medial to the LSA	2nd–4th	Yes, cranial to ARSA	Yes	Regurgitation	Aberrant right subclavian artery	235 days post‐CT, reduction in regurgitation	CR
3 months, female, Chow Chow	Medial and just caudal to the LSA, close proximity to LSA	1st–2nd	Yes, caudal to the ARSA	No	Regurgitation, dyspnea, pyrexia	Bronchopneumonia, septic pleural effusion	CPA during hospitalization	PR
8 years 7 months, female, Irish Setter	Medial and just caudal to the LSA, close proximity to LSA	1st–3rd	Yes, cranial and caudal to the ARSA	Yes	Regurgitation, recurrent gastric dilation, dyspnea, tachypnea	Aspiration pneumonia, septic shock, multi‐organ dysfunction	CPA during hospitalization	PR
2 month, male, French Bulldog	Medial and just caudal to the LSA, close proximity to LSA	3rd–4th	Yes, cranial and caudal to the ARSA	Yes	Regurgitation dyspnea, heart murmur	BOAS, tricuspid dysplasia, ventricular septal defect, atrial septal defect	490 days post‐CT continued regurgitation	PR
6 years 7 months, female, Flat‐coated Retriever	Medial and just caudal to the LSA, close proximity to LSA	1st–3rd	No	Yes	Cough, lethargy, hypoxemia, reduced exercise tolerance	Aspiration pneumonia	932 days post‐CT no regurgitation	PR
7 years 9 months, male, Springer Spaniel	Medial and just caudal to the LSA, close proximity to LSA	1st–3rd	Yes, cranial to ARSA	Yes	Progressive coughing and sneezing	Chronic rhinitis, Aspiration pneumonia	NA	PR
3 years 3 months old, male, French Bulldog	Medial and just caudal to the LSA, close proximity to LSA	1st–4th	No	No	Regurgitation, vomiting coughing, stertor, increased respiratory effort	BOAS	NA	PR
3 years 5 months, male, French Bulldog	Medial and just caudal to the LSA, close proximity to LSA	2nd–3rd	No	No	Regurgitation, stertor, brachycephalic obstructive airway syndrome	BOAS	NA	PR
1 year 8 months, male, French Bulldog	Medial and just caudal to the LSA, close proximity to LSA	2nd–4th	No	No	Regurgitation, dyspnea, stertor, exercise intolerance	BOAS	NA	PR

Abbreviations: ARSA, aberrant right subclavian artery group; BOAS, brachycephalic obstructive airway syndrome; LSA, left subclavian artery; NA, Not available

## 4 Discussion

This study intended to assess the prevalence of ARSA in a large population of dogs and determine any imaging features that could aid in the differentiation between ARSAs which are likely to be of clinical relevance, and those that are likely to be incidental. The prevalence of ARSA in dogs at our hospital was 1.2% (95% CI, 0.6–2.1%). This is similar to that previously reported in the veterinary literature [1, 2], making an ARSA an uncommon finding. Therefore, we reject our hypothesis that ARSAs are more common than previously reported. However, it was the most common anomaly of the aortic arch detected in our population, which is in agreement with other studies [1, 2].

In our population of dogs with an ARSA, this was considered clinically relevant in only one case (2.7%), which was also the only case where further management of the ARSA was performed. As only one case was clinically relevant, we could not assess for a difference in the origin and path of the ARSA between cases where it was clinically significant and those where it was likely incidental. In the relevant case, no other cause of the clinical signs was identified, and clinical signs improved following ligation of the aberrant vessel. It is known that after surgical correction of a VRA, clinical signs do not always completely resolve [5]; however, the fact that this dog was a brachycephalic breed cannot be disregarded. A large proportion of brachycephalic dogs have gastrointestinal signs, including regurgitation and dysphagia [25], in agreement with our findings that a large proportion of dogs presented for thoracic CT at our hospital with regurgitation, were treated for BOAS, including the two dogs with an ARSA that had a history of regurgitation from part one of the study. Brachycephalic dogs presented for treatment of BOAS represented a large proportion of the study population in part one and part two. Although this is likely representative of the true population, this signalment and clinical presentation probably skewed the number of dogs presenting with regurgitation as a clinical sign. In the relevant case, brachycephalic conformation could be a confounding variable despite the lack of other clinical signs attributable to BOAS.

Eight dogs (21.6%) had an ARSA that was considered potentially relevant when assessed as part of the study. Follow‐up information was variable for these patients, with two of them dying during hospitalization, both of which had regurgitation in the clinical history and both of which had a pneumopathy with no other cause for the clinical signs identified. In some of these cases, no regurgitation was mentioned at follow‐up, and in one case, seen for BOAS with a history of congenital cardiac disease, there was continued regurgitation. Without further diagnostics, and due to the study's retrospective nature, the true clinical relevance of the ARSA is difficult to establish. Approximately 5% of humans will develop clinical symptoms associated with an ARSA or other aortic arch abnormalities grouped together and termed arteria lusoria [24, 26]. Some of the clinical signs in humans would be difficult to assess in our patients, such as claudication of the right thoracic limb.

On review of the CT images, esophageal dilation and compression at the site of the ARSA crossing was more common in the clinically and potentially relevant group (100% and 50%, respectively) when compared with the most likely incidental group, where 25% had esophageal dilation and 21.4% had esophageal compression at the site of the ARSA crossing. However, in part one of the study, esophageal dilation was more prevalent in the nARSA group compared with the ARSA group (14.8% and 9.1%, respectively), although no statistical analysis was performed. Assessment of the esophagus under sedation is not considered to be accurate [8]. However, it is often assessed in relation to vascular ring anomalies. The study was unable to confirm the significance of the ARSA in potentially relevant cases based solely on imaging findings. Careful consideration of the appearance of the esophagus is warranted on the clinic floor, considering all other patient factors.

Assessment of the relevance of ARSA is further complicated by the fact that both surgical and medical management can be used successfully to treat the condition. A full review of the dietary history was not available in the medical records. Therefore, some patients with ARSA could already have measures in place that mitigated the clinical signs. In the patient where the ARSA was considered relevant, a VRA was considered due to the positive contrast videofluoroscopic esophagram findings and the patient's frequent regurgitation. However, food and fluid boluses could still pass the region of compression by the ARSA. This has been seen in other studies [10], but it does complicate decision‐making regarding patient management options. We accept our second hypothesis that the number of cases where the ARSA was clinically relevant was low.

In the clinically relevant case, the patient presented to the hospital at two months of age. This contrasts with some case reports where some dogs have presented in later life and in humans where clinical signs are more common after age 40 [24]. This is believed to occur in humans due to the aortic wall becoming atherosclerotic, making it more rigid [24]. Atherosclerosis is rare in dogs, and therefore this etiology and pathophysiology is not an adequate explanation for the development of signs later in life in this species [27]. In one case report in a mature dog, the new onset clinical signs were attributed to a dietary change [19], and in another, it was hypothesized to be in relation to possible acquired bending and/or aneurysmal dilation of the aberrant artery at its origin [17]. Aneurysmal dilation of an aberrant vessel close to its origin, termed a Kommerell's diverticulum, is frequently found in humans with aberrant subclavian arteries and is thought to be part of the congenital anomaly [24]. It has also been reported in cats with ARSA [28, 29]. Interestingly, no aneurysmal dilation at the origin of the aberrant right subclavian artery was seen in our study population. Acute angular bending of the aberrant artery was seen in two cases with an ARSA in our study (5.4%), represented in Figure 3. However, it was only seen in cases where our study considered the ARSA incidental.

As part of the prevalence study, it was found that more male dogs had ARSAs than females, and this difference reached statistical significance (*p* =  .019). In part two of the study, 73% of the cases were male. This finding has not been previously documented. In previous veterinary literature, no sex predilection has been found, although one previous study on VRAs did have a higher proportion of females than males [5]. In a human meta‐analysis on ARSAs, no clear sex predilection was found. However, some papers suggest that females are overrepresented [24, 30]. It is possible that this finding is due to a small sample population of dogs with ARSAs and should be confirmed by further studies. This paper does not provide strong evidence of a breed predilection for ARSA, as the number of dogs representing some breeds was low, particularly for English Setters and Scottish Terriers.

Despite the seemingly minimal clinical relevance for the patient, the finding of an aberrant right subclavian artery could still be relevant for hospitalized patients. Recognition of abnormal anatomy is important for surgical procedures of the thoracic cavity to avoid accidental trauma and the potential for major hemorrhage. Other anatomical abnormalities have been found in humans with ARSA, such as a non‐recurrent laryngeal nerve, which is an important consideration for surgery of the neck [24]. Theoretically, it could be important for anesthetists to be aware of this anomaly, especially during procedures where accidental occlusion of the vessel could lead to abnormal blood pressure readings, such as esophageal endoscopic procedures, and there has been a case report of an ARSA being occluded due to transesophageal echocardiography in a human [31]. In addition, it should be considered prior to any procedure performed via transarterial catheterization of an artery cranial to the aortic arch [32].

The major limitation of the study was the sample population. All cases were from one referral institution, and cases were required to have undergone a thoracic CT, which could have led to selection bias. We tried to mitigate the risk of bias by having the minimum necessary exclusion criteria. The small number of dogs with ARSAs in the study is expected with an uncommon congenital anomaly; however, this means that the statistics performed are likely underpowered. Other limitations include the study's retrospective nature, with the data being fully reliant on information recorded in the patients’ history, the inability to obtain a complete dietary history for the patients, and the timing of regurgitation, be it after eating or after exercise, not being recorded.

In conclusion, while an ARSA was the most common congenital anomaly of the aortic arch in dogs in this study, the prevalence remains low at 1.2% out of 1000 dogs. Only one case of ARSA studied at our institution had clinical signs definitively attributable to the vessel forming a partial vascular ring around the esophagus. No distinctly different origin or path of the ARSA was found in the clinically relevant case compared with the other cases in the study. Although the anomaly is infrequently clinically relevant, it is still an important consideration for patients undergoing procedures such as upper gastrointestinal endoscopy, surgery of the neck or thoracic cavity, and transarterial catheterization.

## List of Author Contributions

### Category 1

- Conception and design: Jones, Klever, Llabres‐Diaz
- Acquisition of data: Jones, Klever, Fouriez‐Lablée, Cordella, Watton, Llabres‐Diaz
- Analysis and interpretation of data: Jones, Klever, Fouriez‐Lablée, Llabres‐Diaz

### Category 2

- Drafting the article: Jones, Klever, Llabres‐Diaz
- Revising the article for intellectual content: Jones, Klever, Fouriez‐Lablée, Cordella, Watton, Llabres‐Diaz

### Category 3

- Final approval of the completed article: Jones, Klever, Fouriez‐Lablée, Cordella, Watton, Llabres‐Diaz

## Conflicts of Interest

The authors declare no conflicts of interest.

## Previous Presentations

This research was accepted for oral presentation at the Pre‐BSAVA EAVDI‐BID Conference meeting in Manchester in March 2024. The authors followed the STROBE reporting guidelines.

## Data Availability

The data supporting this study's findings are available as supplementary files from the corresponding author upon reasonable request.
